# Flexural Strength of Translucent Zirconia Materials Produced with Different Multilayer Technologies: An In Vitro Study

**DOI:** 10.1155/2024/8410101

**Published:** 2024-03-27

**Authors:** Fahad Bakitian

**Affiliations:** Department of Restorative Dentistry, Faculty of Dental Medicine, Umm Al-Qura University, Makkah, Saudi Arabia

## Abstract

**Objective:**

To evaluate the flexural strength of two translucent multilayered zirconia materials produced with different multilayer technologies. *Methodology*. Eighty bar-shaped zirconia specimens were prepared from two different multilayered zirconia materials (IPS e.max® ZirCAD Prime and KATANA™ Multilayered Zirconia HTML) and divided into eight groups (*n* = 10) based on the materials used and the individual layers of the disc for each material: Dentin Prime, Transition Prime, Translucent Prime, Multilayered Prime, Dentin HTML, Transition HTML, Translucent HTML, and Multilayered HTML. The bar-shaped zirconia specimens were cut to include all the layers from translucent to dentin In Multilayered Prime and Multilayered HTML groups. All specimens were fully sintered after cutting from multilayered zirconia discs and subjected to three-point flexural strength test using the universal testing machine.

**Results:**

The specimens made of HTML zirconia material showed significantly (*P* < 0.001) higher flexural strength than those made of IPS e.max® ZirCAD Prime material, with no significant difference (*P* > 0.05) compared to the specimens in the Dentin Prime group. The Dentin Prime specimens had the highest flexural strength (743 ± 116 MPa) compared to those in the Translucent Prime (514 ± 120 MPa), Transition Prime (575 ± 102 MPa), and Multilayered Prime (531 ± 132 MPa) groups. The flexural strength of the specimens from the individual layers of HTML zirconia material was not significantly different (*P* > 0.05) among the Dentin HTML (763 ± 56 MPa), Translucent HTML (791 ± 106 MPa), Transition HTML (816 ± 85 MPa), and Multilayered HTML (793 ± 102 MPa) groups.

**Conclusion:**

Multilayered zirconia materials produced with different yttria contents by layer have lower flexural strength than those produced with gradient shade technology and the same yttria content for each layer. Therefore, various factors such as the type of prostheses, nesting strategies of prostheses within the zirconia disc, and the desired aesthetical requirements should be considered when selecting the multilayered zirconia materials.

## 1. Introduction

Tetragonal zirconia polycrystal stabilized with 3 mol% yttria (3Y-TZP) has been among the most used dental ceramic materials since the nineties [1]. It is a reliable restorative material for making fixed dental prostheses (FDPs) with higher strength and toughness properties compared to other dental ceramic materials [2, 3]. However, due to its high opacity, early-generation zirconia material was specifically used as a framework for FDPs and veneered with aesthetical materials [4]. The inferior optical properties of this zircona material have been attributed to the presence of tetragonal crystal phase [1, 4]. Although tetragonal crystals contribute to material fracture toughness through the phase transformation toughening mechanism, they are birefringent and reduce translucency due to refraction and reflection of light at grain boundaries [4–6]. Conversely, the cubic crystal phase offers improved translucency due to the isotropic properties, which makes the refractive index unaffected by the direction of light travel [7–9]. Nonetheless, zirconia material with a substantial amount of cubic crystals would benefit less from the phase transformation toughening, making it less resistant to fracture [7–9].

Recently, new zirconia materials have been developed with enhanced translucent properties, allowing full-contour (monolithic) FDPs to overcome the clinical issues of veneer chipping and fracture [10, 11]. The improved translucency was achieved by altering the chemical composition by using a higher yttria content compared to the traditional zirconia, which results in more of the optically isotropic cubic phase [7–9]. Nevertheless, such attempts to raise the translucency of zirconia materials can result in inferior mechanical properties in terms of fracture strength and toughness [7–9]. To overcome this issue, manufacturers have developed new multilayered zirconia materials intended for monolithic FDPs.

Translucent multilayered zirconia products are different from each other based on the implemented multilayer technology, with some products having the same chemical composition by layer but with a gradient shade technology [12, 13]. In this technology, zirconia materials have different color additives by layer to gradually increase the translucency from the cervical area to the incisal area of the restoration. The multilayering effect is achieved by controlling the color additives in each layer, and the differences in translucency are achieved indirectly by reducing light transmittance. The translucent layer is free of color pigments, while the opaque layer has the highest saturation of color pigments. It is important to note that achieving a balance between mechanical and optical properties in multilayered zirconia materials using grade shade technology can be challenging. This balance is highly dependent on the yttria content in the material [7]. For instance, while multilayered zirconia with 3% mol yttria content produced with grade shade technology has high strength, its translucency may be low. Conversely, using the same technology, multilayered zirconia with 4% or 5% mol yttria contents may have high translucency but limited flexural strength [8, 9].

To address this issue, other multilayered zirconia materials have been developed featuring different chemical compositions in terms of yttria contents in each layer, resulting in a material that offers both pleasing aesthetics and high strength [14]. In this multilayer technology, the upper half of the manufacturing disc, which represents the incisal area of the restoration, is made of translucent zirconia material with a high yttria content. The lower half, which represents the cervical area, is made of low translucent zirconia with 3 mol% yttria content; thus, the mechanical properties of these materials are expected to be different by layer [7, 14]. Therefore, dental technicians need to have enough knowledge and carefully handle those translucent multilayered zirconia materials during the fabrication process using a computer-aided design/manufacturing (CAD/CAM) system.

There are a variety of translucent multilayered zirconia materials made with different multilayer technologies in the dental market, and more research is required to evaluate the effect of those technologies on the flexural strength of the zirconia material. Therefore, the present study evaluated the flexural strength of two translucent multilayered zirconia materials produced with different multilayer technologies. The null hypotheses tested were that there is no difference in flexural strength values of two translucent zirconia materials produced with different multilayer technologies and that there is no difference in flexural strength values of the individual layers of those two multilayered materials.

## 2. Methodology

### 2.1. Sample and Materials

**Figure 1:**
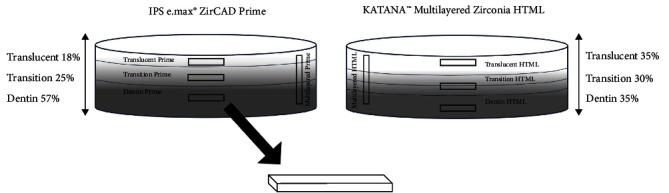
The illustration shows the study groups and how the specimens were cut from the individual layers of the two multilayered zirconia discs. The rectangles within the discs represent the eight study groups and the numbers show the thickness of each layer in percentage.

Eighty bar-shaped zirconia specimens were prepared from two different partially sintered multilayered zirconia discs and divided into eight groups (*n* = 10) based on the multilayered zirconia materials used and the individual layers of the discs (Figure 1): Dentin Prime, Transition Prime, Translucent Prime, Multilayered Prime, Dentin HTML, Transition HTML, Translucent HTML, and Multilayered HTML. The bar-shaped zirconia specimens were cut to include all the layers from translucent to dentin in the Multilayered Prime and Multilayered HTML groups.

Two types of translucent multilayered zirconia materials produced with two different multilayer technologies were used in this study (Table 1): IPS e.max® ZirCAD Prime (Ivoclar, USA) and KATANA™ Multilayered Zirconia HTML (Kuraray Noritake Dental, EU). The ZirCAD Prime disc measures 16 mm in height with a 98.5 mm diameter and comprises three layers with different yttria contents based on the manufacturer data: The incisal (translucent) layer represents 18% of the total disc height, the transition layer represents 25% of the disc height, and the dentin (body) layer represents 57% of the disc height (Figure 1). The Multilayered Zirconia HTML disc measures 18 mm in height with a 98.5 mm diameter and comprises four layers of zirconia with a gradient shade technology: The enamel (translucent) layer represents 35% of the disc height, two transition layers represent 30%, and the dentin layer represents 35% (Figure 1).

The zirconia specimens were prepared by one individual based on the European standard ISO 6872:2015 using an automatic precision cutting machine (Struers Secotom-60, Struers, USA). The diamond saw was used to cut the specimens into rectangular-shaped bars directly from the zirconia discs. The zirconia specimens were then finished using a grinder/polisher machine (Minimet, Buehler, Lake Bluff, IL, USA) with 600-grit paper until they reached the standard dimensions of 1.2 ± 0.2 mm thickness by 4.0 ± 0.2 mm width and 15.0 ± 0.2 mm length. Subsequently, they were polished with 1200 and 2400-grit paper.

### 2.2. Sintering Procedures and Flexural Strength Test

The zirconia specimens were sintered using a calibrated ZirkonZahn dental ceramic furnace (ZirkonZahn; Zirkonofen 700 Vakuum, Zirkonzahn GmbH, Gais, Austria) directly after cutting from each layer of the multilayered zirconia discs. The sintering conditions were based on the standard sintering programs following the manufacturer's instructions (Table 2). Before the bending test, the specimens were cleaned for 10 min using ultrasonic cleaning in distilled water.

A static three-point bending test based on ISO 6872 was performed using a universal testing machine (Zwick/Roell type: Xforce HP) to determine the flexural strength of each layer of the multilayered zirconia discs. Each zirconia specimen was placed on two supporting beams measuring 1.5–5 ± 0.2 mm in diameter. The zirconia specimens were loaded using the universal testing machine centrally at a crosshead speed of 1 mm/min until fracture occurred. The span was set at 13.0 mm. Specimens were tested dry at room temperature. The load at fracture was recorded in *N* and the flexural strengths were calculated in MPa automatically as recommended in the standard and based on the following formula:

(1) $$\begin{array}{c} \sigma =3Nl/2bd^{2}, \end{array}$$

where *σ* is the flexural strength (MPa), *N* is the fracture load (in Newton), *l* is the distance between the supports (in mm), *b* is the width of the specimen (in mm), and *d* is the thickness of the specimen (in mm).

### 2.3. Statistical Analysis

The normal distribution and homogeneity of the data were verified by the Kolmogorov–Smirnov test and Levene test. Then, the differences in the flexural strength among the tested groups were analyzed using a two-way analysis of variance (ANOVA), followed by Tukey's post hoc test (IBM SPSS Statistics 22; SPSS Inc., Chicago, IL, USA). Results are considered statistically significant at *P* ≤ 0.05. The power analysis was based on previous research with a comparable study design to detect differences between zirconia-based specimens [14, 15].

## 3. Results

**Figure 2:**
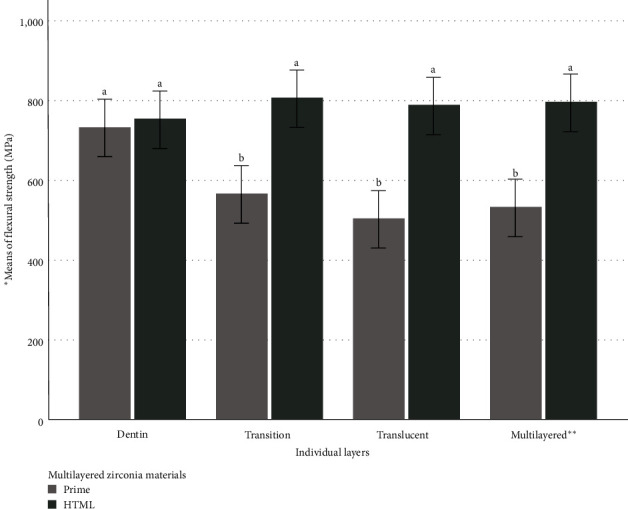
Differences in mean flexural strength (MPa) among the groups with two different multilayered zirconia materials. *Notes*.  ^*∗*^Means with the same letter ^a,b^ are not significantly different in flexural strength (*P*  > 0.05).  ^*∗∗*^Multilayered groups represent the groups with specimens involving all layers of the zirconia discs.

The three-point flexural strength data and significance levels of all groups are shown in Figure 2. There were significant differences in flexural strength values among the groups according to the two multilayered zirconia materials with different multilayer technologies used in the study (*P* ≤ 0.05). Generally, the specimens made of HTML zirconia material had significantly (*P* < 0.001) higher flexural strength than those made of IPS e.max® ZirCAD Prime material but were not significantly different (*P* > 0.05) compared to the Dentin Prime group.

**Figure 3:**
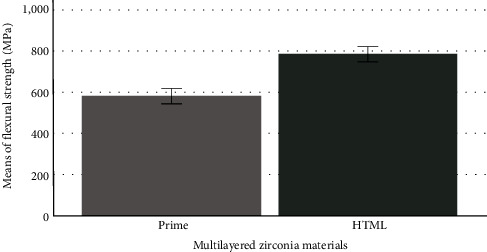
Comparison of flexural strength of the two multilayered zirconia materials with different multilayer technologies.

Figure 3 compares the flexural strength of the two multilayered zirconia materials with different multilayer technologies regardless of the individual layers. The Dentin Prime specimens had significantly (*P* < 0.05) highest flexural strength (743 ± 116 MPa) compared to those in Translucent Prime (514 ± 120 MPa), Transition Prime (575 ± 102 MPa), and Multilayered Prime (531 ± 132 MPa) groups. Conversely, the specimens from the individual layers of HTML zirconia material were not significantly different (*P*=0.437) in flexural strength from the Dentin HTML (763 ± 56 MPa), Translucent HTML (791 ± 106 MPa), Transition HTML (816 ± 85 MPa), and Multilayered HTML (793 ± 102 MPa) groups.

## 4. Discussion

The study results indicated that the multilayered zirconia material with different yttria contents by layer significantly differed in flexural strength values compared to the multilayered zirconia material with gradient shade technology; hence, the first null hypothesis was rejected. However, the study showed no significant differences in the flexural strength of individual layers of the multilayered zirconia material with gradient shade technology compared to individual layers of the other material with varying yttria contents. Therefore, the second null hypothesis, assuming that there is no difference in the flexural strength values of the individual layers of the two multilayered zirconia materials tested in this study, was partially rejected.

Even though newly developed multilayered zirconia materials are more translucent than traditional zirconia materials stabilized with 3% mol yttria [4, 7–9], the balance between strength and translucency properties is a significant factor for the success of those materials. Manufacturers have implemented different technologies for enhancing the material translucency to mimic the optical properties of natural teeth. This study evaluated the flexural strength of two multilayered zirconia materials made with different technologies. The IPS e.max® ZirCAD Prime is a multilayered material with two zirconia materials stabilized with different yttria contents (3% and 5% mol) in one disc, each material in one layer with an intermediate transition layer containing a mix of the two between them to facilitate a gradual change in the chemical composition. Conversely, KATANA™ Multilayered Zirconia HTML consists of only translucent zirconia material stabilized with 5% mol yttria in each layer but with varying color additives to control the translucency. Regardless of the implemented technology, the preferable technology is the one that better balances the strength and translucency properties.

The present study revealed statistically significant differences in the flexural strength of the individual layers of the ZirCAD Prime disc, with the dentin layer having the highest flexural strength. This result can be explained by the fact that the dentin layer consists of the traditional zirconia material stabilized with 3% mol yttria which has a high strength and tough metastable tetragonal crystal phase due to transformation toughness [1, 5, 6]. In this mechanism, surface stresses at the tips of preexisting cracks encountered during fabrication induce the transformation of tetragonal crystals into monoclinic crystals, thereby increasing the volume and compressing the surface to inhibit further crack propagation and increase the strength and toughness properties [5, 6]. In contrast, the translucent layer of the ZirCAD Prime material had the lowest flexural strength values compared to the other layers. This layer consists of highly translucent zirconia material stabilized with a higher yttria content (5 mol%) compared to traditional zirconia. The increase in yttria content results in more cubic phase formation at the expense of the metastable tetragonal phase at the microstructural level [7, 8, 16, 17]. As previously reported, the latter influences the ability for transformation toughening of those cubic containing zirconia materials [16, 17]. Therefore, the strength and toughness properties of the translucent layer might be negatively influenced by using a higher yttria content compared to the dentin layer. Moreover, the flexural strength of the transition layer of the ZirCAD Prime material was higher but not significantly different from the translucent layer. The higher strength properties of this layer compared to the translucent layer might be achieved by the presence of the tough zirconia material with 3 mol% yttria.

The other multilayered zirconia material (KATANA HTML) tested was not statistically significantly different in mean flexural strength among the individual layers, indicating that there are few differences in the mechanical properties throughout the different layers. This may be attributed to the similar yttria content used in each layer of this multilayered zirconia material, and the translucency properties differed only by the amount of color additives. These results are in line with previous studies that reported no significant differences in the flexural characteristics of zirconia materials subjected to different coloring procedures [18, 19]. Conversely, a previous study reported that more color additives with different amounts of iron and titanium in the dentin layer resulted in an increased monoclinic content after low-temperature degradation [20]. The high monoclinic content has a deteriorating effect on the mechanical properties and results in an unstable material that experiences crack development under severe conditions. In this study, the flexural strength of the dentin layer of the KATANA HTML material was lower but not statistically different compared to other layers. Further investigations are needed to understand the low-temperature degradation behavior of the individual layers of multilayered zirconia materials made with gradient shade technology.

For fabricating monolithic ceramic restorations, silicates and zirconia are two commonly used materials in dentistry [10, 21, 22]. Vichi et al. [23] conducted a study on the flexural strength values of silicates and found that their strength values ranged from 127.65 to 350.88 MPa. These materials are suitable only for single-unit prostheses and for 3-unit prostheses not involving molar restoration, as they meet the flexural strength values required for classes 1, 2, and 3 of ISO standard 6872:2015 [24]. Compared to the findings of this study on zirconia materials, it was observed that multilayered zirconia materials for monolithic restorations showed higher strength values than silicates. All of the multilayered zirconia materials tested in this study achieved flexural strength values higher than 200 MPa of what is required for classes 1, 2, and 3 of ISO standard 6872:2015. Additionally, zirconia materials with a gradient shade technology demonstrated even higher flexural strength values, making them suitable for clinical indications of 3-unit prostheses that involve molar restoration, based on class 4 of the ISO standard 6872:2015 [24].

In dental laboratories, the tested multilayered zirconia materials are milled from presintered discs to fabricate FDPs using CAD/CAM technology [25]. This technology allows the dental technician to place the intended prosthesis within the multilayered zirconia disc by moving it up and down using the CAM software. This study clarified the significant effect of different yttria contents on the flexural strength of the individual layers of the multilayered ZirCAD Prime material compared to the KATANA HTML material with similar yttria contents in each layer. Although the ZirCad Prime offers high aesthetical outcomes with varying translucency for each layer, it might be more sensitive to the placement procedures of dental prostheses in the disc than the KATANA HTML material with a gradient shade technology. Those procedures, therefore, critically influence the clinical success of dental prostheses, especially if the weak parts of the prosthesis, such as the connectors, are placed in transition or translucent layers [13, 26–28]. Further studies are needed to evaluate the influence of these placement procedures on prostheses made of multilayered zirconia materials with different yttria contents.

One of the limitations of laboratory studies evaluating multilayered zirconia materials is the challenge of standardizing the preparation of the test specimens made of the different layers. The position of the specimens prepared from each layer of the presintered disc may have differed, which could contribute to significant differences that affect the study results. Choosing the appropriate standard for conducting the test is crucial for meeting the study's aims. Additionally, comparing test results with other studies using the same test protocols is important. In this study, all specimens were prepared following the ISO 6872:2015 before sintering.

The laboratory study that evaluates dental restorative materials is recommended to be clinically relevant to mimic the clinical failures of dental restorations [13, 27, 29]. The specimen shape might be one of the limitations of this study. Although the specimen dimensions are based on ISO standards, the strength and fracture behavior of the different multilayered translucent zirconia materials tested should be further investigated with anatomically shaped specimens, such as crowns and bridges. Moreover, other material properties should be investigated such as elemental composition, grain sizes, microhardness, and fracture toughness of the individual layers of translucent zirconia materials made with different multilayer technologies.

## 5. Conclusion

Within the limitations of the study, the following can be concluded: Translucent zirconia materials produced with different multilayer technologies are relatively different from each other regarding their chemical composition. Multilayered zirconia materials produced with different yttria contents by layer have lower flexural strength than those produced with a gradient shade technology and the same yttria content for each layer. Therefore, various factors such as the type of prostheses, nesting strategies of prostheses within the zirconia disc, and the desired aesthetical requirements should be considered when selecting the multilayered zirconia materials.

## Figures and Tables

**Table 1:** Zirconia materials made with two implemented multilayer technologies.

Materials used	Multilayer technology
IPS e.max® ZirCAD Prime	Three layers of zirconia materials with different yttria contents by layer
KATANA™ Multilayered Zirconia HTML	Four layers of one zirconia material with a gradient shade technology (same yttria content by layer)

**Table 2:** Sintering parameters for the two multilayered materials used.

Materials used	Heating phases	Heating rate (°C/min)	Sintering temperature (°C)	Holding temperature (°C)	Holding time (min)	Cooling rate (°C/min)	Total sintering time
IPS e.max® ZirCAD Prime	Phase I	10	900	900	30	10	9 hr 50 min
	Phase II	3.3	1,500	1,500	120	8.3	

KATANA™ Multilayered Zirconia HTML	One phase	10	1,550	1,550	120	10	7 hr

## Data Availability

The data that support the findings of this study are available from the corresponding author upon reasonable request.
